# A Grounded Theory of the Lived Experiences of People with Pancreatic Cancer in Northern Ireland: Study Protocol

**DOI:** 10.3390/healthcare13212779

**Published:** 2025-11-01

**Authors:** Lana Cook, Gillian Prue, Susan McLaughlin, Gary Mitchell

**Affiliations:** 1 School of Nursing and Midwifery, Queen’s University Belfast, Medical Biology Centre, 97 Lisburn Road, Belfast BT9 7BL, Northern Ireland, UK; g.prue@qub.ac.uk (G.P.); gary.mitchell@qub.ac.uk (G.M.); 2 Northern Ireland Pancreatic Cancer (NIPANC), 384 Belmont Road, Belfast BT4 2NF, Northern Ireland, UK

**Keywords:** pancreatic cancer, psycho-oncology, psychosocial, experiences, grounded theory, study protocol, photovoice, qualitative research

## Abstract

**Background/Objectives**: Pancreatic cancer remains highly fatal, often diagnosed late with poor prognoses and worse psychological quality of life compared to other cancers. Globally, it is the twelfth most common cancer but the sixth leading cause of cancer-related deaths, with actual 5-year survival rates below 5%. Northern Ireland’s outcomes are among the worst, yet research on people’s experiences across the illness trajectory is scarce. Consequently, the unique needs of people with pancreatic cancer are poorly understood. It is crucial we develop deeper understanding of the entire pancreatic cancer journey to address this. This study aims to explore the lived experiences of people diagnosed with pancreatic cancer in Northern Ireland and generate a theory that explains their journeys, from pre-diagnosis through to survivorship or end of life. **Methods**: This study will adopt a grounded theory approach, incorporating multiple qualitative data generation methods: semi-structured interviews with patients and care partners, and focus groups with professionals. An optional photovoice (participatory photography) method will be offered to participants. Theoretical sampling principles and constant comparative analysis will guide recruitment, data collection, and analysis to ensure the explanatory theory is rooted in participants’ lived experiences. **Conclusions**: Establishing a holistic, in-depth understanding of people’s pancreatic cancer journeys will enable us to better comprehend, anticipate, and meet their needs. A theory grounded in empirical data about lived experiences can inform priorities for future care, support services, policy, and research, and contribute to the development of support interventions that help people to maintain the best possible quality of life, whether during a short-term, terminal illness; treatment journey; long-term symptom management; or survivorship.

## 1. Introduction

Pancreatic cancer remains highly fatal due to a lack of progress in screening, early detection methods, and available treatment options [1]. Consequently, mortality rates remain extremely high; recent real-world analyses suggest that actual five-year survival rates are below 5% overall and below 1% for inoperable cancers [2]. It is now the twelfth most common and sixth deadliest cancer worldwide, with an estimated 510,992 cases and 467,409 deaths in 2022 [3]. Although prognosis is poor in general, survival rates vary significantly by region, country, and healthcare system. In high-income economies, actuarial five-year survival rates (modelled estimates based on population data, accounting for other causes of death [4]) have increased modestly; the most recent prevalence data shows that Australia’s have reached 14.6%, Ireland’s 9.6%, and the UK’s 7.5% [5]. Northern Ireland’s were only 6.2% in the same period [6], but recent cancer registry data reports an increase to around 8% for people diagnosed from 2013 to 2017 [7]. According to CONCORD-3 data, Northern Ireland (NI) ranks 32nd of out 36 comparable countries for five-year pancreatic cancer survival [6] and around 291 people are diagnosed annually [7]. These inequities may reflect differences in gross domestic product (GDP) per capita, investment in health infrastructure, and the availability of cancer care services. They may also be linked to how care pathways enable timely access to diagnosis and surgery, both of which strongly influence survival outcomes [8]. An Optimal Care Pathway for people with pancreatic cancer has recently been developed and implemented in NI to address these challenges [8].

Supporting people to maintain the best possible quality of life (QoL) for as long as they can is essential [9], and understanding the unique challenges faced by people living with pancreatic cancer is critical for the development of effective support interventions that meet their needs and enhance their wellbeing—be that through a short-term, terminal illness; their journey with treatment(s); or the long-term impact of symptom management [10]. There are several studies which have examined the symptom burden associated with the advanced stage common at diagnosis and the treatment options available; these relate to pancreatic exocrine insufficiency, cancer anorexia-cachexia syndrome, diabetes, and cancer progression [11,12,13,14,15,16,17,18].

Furthermore, we know that when compared to other cancers, people with pancreatic cancer face similar reductions in physical and social QoL, but worse psychological QoL [19]. The significant worsening of health-related QoL can manifest in cognitive decline, comorbid depression, distress, anxiety, and reduced coping with the disease; this is attributed to its low curability and survival [13,20], and ineffective assessment, multi-disciplinary management, and support for symptoms of pancreatic exocrine insufficiency [10,18,21,22], pain, and fatigue [14,23].

Of note, a recent qualitative study [12] identified people with pancreatic cancer were impacted post-surgically for significantly longer than thought, perhaps due to previous use of quantitative surveys rather than in-depth interviewing. Authors highlighted the need for more qualitative research with this population [12]—a need reflected by the selective aspects of the pancreatic cancer journey explored in the literature. Most focuses on specific aspects of patients’ experiences, such as neoadjuvant chemotherapy [13], post-surgical symptoms and outcomes [10,24], or information sharing and communication around decision-making and treatment options [12,25,26,27]. This omits many groups of people with pancreatic cancer—particularly those who are not candidates for these treatments and therapies. Literature which seeks to capture the journey from pre-diagnosis to survivorship and/or end of life is scant. Furthermore, no studies were discovered from NI and few from the United Kingdom (UK).

Therefore, this proposed study seeks to explore the experiences of people with pancreatic cancer more broadly and establish holistic, in-depth understanding of the process of people’s pancreatic cancer journeys across the illness trajectory. It will be undertaken in collaboration with our charity partner, NIPANC (Northern Ireland Pancreatic Cancer).

## 2. Aim, Research Question, and Objectives

This study aims to explore the experiences of people diagnosed with pancreatic cancer in NI and, using grounded theory methodology, generate a theory to explain the process of their journeys. This will answer the following research question: How do individuals experience pancreatic cancer across the trajectory in NI, from pre-diagnosis through survivorship or the end of life?

The main objectives are as follows:

1. To conduct a systematic review of empirical literature on the psychosocial aspects of receiving, living with, surviving, or dying from a pancreatic cancer diagnosis. This will contextualise the study within existing research and inform the initial topic guide for interviews.
2. To explore the experiences of people diagnosed with pancreatic cancer, through semi-structured interviews (SSIs) with people diagnosed with pancreatic cancer.
3. To explore the perspectives of care partners and family members through SSIs, both to gain additional insight into the experiences of people with pancreatic cancer, and to act as proxies for the experiences of people who are deceased.
4. To explore the perspectives of healthcare professionals and others who give care, support, and advocacy to people diagnosed with pancreatic cancer, providing additional insight and data triangulation.

## 3. Materials and Methods

### 3.1. Study Design and Methods

This study will adopt grounded theory (GT) methodology [28,29], incorporating multiple qualitative data collection methods: SSIs and focus groups (FGs) supplemented by an optional photovoice method [30].

GT was chosen since little is known about the lived experiences of people with pancreatic cancer across the illness trajectory and because an intended outcome of this study is the generation of theory which can explain these journeys [31]. The inductive, systematic approach of GT enables researchers to gain deeper comprehension of participants’ experiences and facilitates the development of theory directly from the data [32,33] through an iterative process of simultaneously collecting and analysing data [28,29]. The GT will be guided by Chiovitti and Piran’s recommendations on rigour in GT research [34].

Photovoice is a participatory research method that was chosen as an optional supplementary approach to enrich data collection, meaning that participants can choose to participate in an interview with or without engaging with the photovoice component. Those who opt in will be asked to take a photograph which represents an aspect of their (loved one’s/patients’/client’s) experiences with pancreatic cancer and to share this with the primary researcher prior to discussing it at interview. Photovoice empowers participants by inviting them to capture and voice their experiences and perspectives through sharing their own photographs. Integrating photovoice has three intended benefits for this research:

1. It will alleviate some perceived power imbalances in the researcher–participant relationship by giving participants more control over how experiences are represented, both visually (by sharing a photograph) and then verbally (by clarifying what their photographs represent and captioning them) [35,36]. This empowers participants to co-create data, and is particularly important for people with pancreatic cancer, as they may not be able to participate in later member checking events.
2. It will promote dialogue and knowledge about the strengths and concerns of people living with pancreatic cancer, supporting discussions of concepts that are more difficult to put into words alone, and enabling researchers to obtain deeper insights into the pancreatic cancer journey by combining rich visual and verbal data.
3. Where participants’ give explicit consent for their deidentified photographs to be shared, their voices will have the potential to reach policy makers and the wider public, as photographs (and their associated captions) have the potential to improve the approachability of research findings and connect with academic and non-academic audiences.

The photovoice method is informed by Wang and Burris [30] and Wang et al.’s work [37] and guided by the process put forward by Evans-Agnew et al. [38].

### 3.2. Study Phases

The study will comprise four phases. Phase 1 is a qualitative systematic review of existing empirical literature on the psychosocial aspects of the experiences of people with pancreatic cancer. This will identify research gaps, inform initial interview topic guides, and seat the study within its wider context. The review protocol is registered with PROSPERO (CRD42025635882).

This study protocol will focus on the subsequent three phases of data collection:

- **Phase 2**: In-person, SSIs with people diagnosed with pancreatic cancer to explore their experiences.
- **Phase 3**: In-person, SSIs with care partners of people diagnosed with pancreatic cancer to explore their perspectives and insights into the experiences of people with pancreatic cancer.
- **Phase 4**: FGs with professionals who have experience providing care, advocacy, and/or support to people diagnosed with pancreatic cancer, to gather insight on the care, support needs, and perceived barriers to care and support for people with pancreatic cancer.

**Figure 1:**
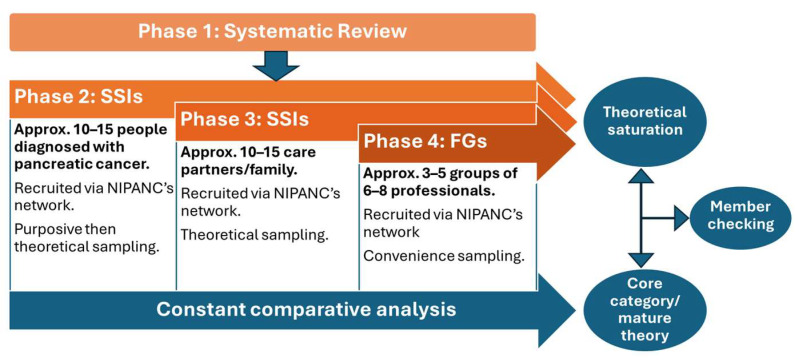
Phases of study.

Integrating and triangulating data from multiple sources through each phase of the study will enhance its credibility, validity, and theoretical grounding [39,40]. Simultaneous data collection will take place during Phases 2, 3, and 4, but the commencement of each phase will be staggered as per Figure 1. Data types will comprise the following:

1. Audio recordings (SSIs, FGs, and voice memoing post-interviews)
2. Written, physical, and digital materials (written memos, observation notes, and photographs (physical or digital) provided by participants who opt for the photovoice method if they have consented to their incorporation in data analysis.

The resources required, and process followed, for each phase of data collection is outlined in Table 1. Whether participants opt to use photovoice or not will not preclude them from being interviewed on an individual or group basis.

### 3.3. Setting

The research settings will be participants’ own homes and non-NHS site public spaces across NI that are preferred by participants and provide sufficient privacy, comfort, and convenience for data collection. Ideally, these should be quiet to prevent challenges in recording audio [41] and provide enough space for FGs.

### 3.4. Sample Size

The sample size will be determined by theoretical saturation [28]; this means that once no new insights emerge from sampling and the existing data/theory is adequate and the categories that comprise the theory have no gaps left in them, theoretical saturation has been reached. Therefore, the data drives the sample size, and it is not a fixed number. However, rough estimates have been given in this protocol based on other qualitative research and grounded theory studies. The sample sizes of GTs in similar populations varies [42,43,44,45], but we expect approximately 20–30 participants for individual interviews (10–15 people with pancreatic cancer and 10–15 care partners) and approximately 18–40 participants for FGs (3–5 groups of 6–8 professionals).

### 3.5. Expert Reference Group

An expert reference group (ERG) will be established to provide oversight throughout the study, meeting online 2–3 times each year. This steering will enhance methodological rigour and ensure the planned study is appropriate and robust by highlighting any gaps or areas needing addressed to achieve its aim of generating a theory which explains the process of the pancreatic cancer journey in NI. Involving a network of expert stakeholders in the study will also lend itself well to dissemination and the translation of research findings into practice. This will comprise approximately 5–10 experts from the following areas:

- Researchers with expertise in pancreatic cancer and/or other less survivable cancers.
- Researchers with expertise in grounded theory or another qualitative methodology.
- Hepato-pancreato-biliary (HPB) or upper gastrointestinal specialists, such as clinical nurse specialists, dieticians, surgeons, and gastroenterologists.
- People with lived experience of pancreatic cancer as either survivors or relatives.
- Representatives of pancreatic cancer charities.

### 3.6. Interview Guide

Theoretical sampling can be achieved in various ways within a project—beyond the sampling of participants to the actual data collection process itself [46]. In this study, theoretical sampling will be achieved during interviews by “steering questions in the direction of the emergent theorizing” ([46], p. 954). Therefore, the initial interview guide used for interviews will be brief and concise, guided by Foley et al. who suggest that it “should be so succinct that going out to do the first few interviews… the researcher should be wondering—is the interview guide sufficient to go by?” ([47], p. 3). This is to avoid hurried, surface-level interviews that, whilst covering a broad number of topics, may not provide enough depth, and “prove conceptually unsatisfactory... [making] theoretical sampling more difficult or even impossible.” ([47], p. 3). Interview guides should also evolve in tandem with the lines of inquiry which also direct the sampling of participants ([46,47]).

### 3.7. Phase 2: People Diagnosed with Pancreatic Cancer

Participants and eligibility criteria

Participants will include people who have received a pancreatic cancer diagnosis at any time. Full eligibility criteria are outlined in Table 2; these have been designed in partnership with a local charity that represent people who have lived with, or supported people with, pancreatic cancer. Gatekeepers will support recruitment directly from their support groups and screen potential participants for these criteria.

Sampling method

In line with GT, sampling will initially be purposive and then shift to theoretical sampling in response to emerging insights from the data [29,31,50]. This means that sampling will be strategic and dynamic, enabling researchers to refine and validate the emerging theory [28,33]. Purposive sampling will seek to gather a diverse range of participants to ensure a wide variety of demographic characteristics such as age, ethnicity, and gender; and contextual factors such as location (rural versus urban), the healthcare Trust people live(d) and receive(d) care within, the stage of cancer at diagnosis, and operable versus inoperable cancers. The data collected from this initial sample of participants will be coded and analysed before subsequent participants are sampled. At this point, sampling will move to a theoretical sampling approach. If we do not recruit enough participants through NIPANC’s network, we will employ a snowball sampling technique to reach intended participants.

Recruitment

Recruitment will take place primarily through social media and our charity partner’s newsletter, support group meetings, and events. Their network includes people diagnosed with pancreatic cancer (historically or currently).

People interested in participating will be asked to contact the primary researcher (LC) directly via an expression of interest form or to consent to their details being passed to the primary researcher (LC) if they have directly expressed interest to gatekeepers. Potential participants will be contacted by the primary researcher (LC) via email and telephone to provide comprehensive information on what’s involved, the aim of the study, and data collection procedures (via participant information sheets). Speaking via telephone will also give potential participants the opportunity to ask questions about the research. Subsequently, the primary researcher (LC) will enrol interested participants into the study.

Data collection

The primary method of data collection will be in-person, SSIs to gather rich, in-depth data [51]. These will be recorded using digital voice recorders and observation notes, and the primary researcher (LC) will write memos in line with GT [28,52]. Participation in photovoice will be optional for participants. Interviews will either follow a traditional SSI approach or one blended with the photovoice method.

### 3.8. Phase 3: Care Partners of People Diagnosed with Pancreatic Cancer

Participants and eligibility criteria

Participants will include care partners of people who have received a pancreatic cancer diagnosis at any time. Full eligibility criteria are outlined in Table 2.

Sampling method

In Phase 3, we will continue with theoretical sampling in response to emerging concepts from the data analysis and developing theory [29,31,52].

Recruitment

Recruitment for care partners will follow the approach outlined in Phase 2, but recruitment materials will specify care partner participants and explain what this means. Our charity partner’s network includes care partners (family and friends), predominantly bereaved, of people diagnosed with pancreatic cancer.

Data collection

Data collection will follow the approach outlined in Phase 2.

### 3.9. Phase 4: Professionals and Advocates with Insight into Pancreatic Cancer Journeys

Participants and eligibility criteria

Participants will include professionals, advocates, and support personnel with insight into people’s pancreatic cancer journeys, such as HPB clinical nurse specialists, surgeons, hepatologists, HPB advanced practitioner dieticians; critical care nurses and step-down care staff in hepatology; members of the palliative care team; and district nurses and other community staff); counselling organisations; pastoral support and faith groups; and support services within the voluntary sector. Full eligibility criteria are outlined in Table 2.

Sampling method

For practical reasons, convenience sampling will be used in Phase 4, and FGs will likely take place preceding or following on from events hosted by or related to our charity partner, since the participant population will already be gathered. This is with a view to respecting participants’ time commitments and limiting no-shows. However, convenience sampling will be complemented by purposive and theoretical sampling principles, as efforts will still be made to purposively sample participants with varied perspectives, backgrounds, and insights into the lived experiences of people diagnosed with pancreatic cancer.

As theory is developed from the analysis of this data, theoretical sampling will take place for subsequent FGs (and potentially mini-group or individual interviews if more feasible or practical) to test emerging theory, such as by sampling professionals who have clinical experience of working with people diagnosed with pancreatic cancer within an emergency department to test concepts around lived experiences relating to diagnosis. Where this is not possible, snowball sampling may be used to reach the appropriate professionals.

Recruitment

Recruitment materials for Phase 4 participants will be distributed in the same channels as for Phases 2 and 3, but also throughout professional associations and relevant online forums. Our charity partner’s network includes professionals and advocates.

Data collection

Focus groups have been chosen for interviews with professionals and advocates due to their ability to identify knowledge that is shared anecdotally amongst people with insight into the experiences of people with pancreatic cancer, but which might not otherwise be captured [53]. If Phase 4 participants choose not to use photovoice, it is likely they will still be part of a focus group where other participants do bring photographs to represent their insights into patients’, clients’, or service users’ experiences of living with pancreatic cancer. In these instances, other participants’ photographs can serve as photo-elicitation artefacts for participants who did not bring their own photographs.

### 3.10. Data Analysis

In line with the fundamental components of GT, a process of concurrent data collection and analysis will be implemented [31]. This analysis method is called constant comparative analysis and will take place iteratively from the beginning of data collection until theoretical saturation has been reached and a mature grounded theory has been developed. Data from interview transcripts will be coded and integrated with visual and contextual data from photovoice contributions. Coding will be carried out manually by the primary researcher (LC) and seeks to identify patterns in participants’ contributions and inform the development and later refinement of categories, concepts, and theories [54]. It will proceed through several stages: initial open, selective, and then theoretical coding [28,31].

Constant comparative analysis will proceed within each phase and across phases simultaneously, meaning that codes and emerging categories will be iteratively compared for similarities and divergence. Categories will then be integrated and refined further until a core category and/or a mature and comprehensive framework of theory has been developed that encapsulates all categories. Once the research team agrees that theoretical saturation has been reached, the process of data collection and data analysis will conclude. Node structures in NVivo will be utilised to manage and organise codes and conceptualise categories throughout this process.

Memoing and reflexivity

Throughout the research process, memoing will capture initial impressions, observed patterns, and emerging insights within each phase, as well as observed patterns and connections across phases. Memos will refer to theoretical, methodological, and operational aspects of the study, providing an audit trail of the decision-making process that enhances dependability and confirmability [55]. Memos will be reviewed regularly to inform the process of reflexivity, which is defined as an “active, systematic process used by the researcher to develop insight into their work and guide future actions” ([31], p. 35). Through exercising reflexivity, researchers strive to balance their theoretical understanding with an awareness of the biases they hold that could influence their decision-making [38]. Practicing reflexivity from the outset of the research will help to mitigate the potential impact of bias originating from personal, professional, and educational experiences, improving trustworthiness in line with Lincoln and Guba’s framework [55].

Member checking

Member checking will be carried out at multiple time points throughout the study to validate findings and ensure their accuracy and credibility [55]. Participants’ feedback during these time points will be documented and integrated into the analysis if appropriate, with the primary researcher (LC) reflecting on how this feedback influenced emerging theory. If necessary, further sampling and constant comparative analysis will be carried out until the developed theory resonates with participants’ experiences and insights. Member checking has the potential to be a therapeutic or distressing process, and participants in this study are likely to then be at a different stage of their journeys with pancreatic cancer than they were during data collection—whether that is their journey with diagnosis, treatment, bereavement, or processing the subsequent death of a loved one [56]. Therefore, member checking post-analysis will seek to ensure that the theory emerging from the data does resonate with participants, and that the exercise of member-checking is meaningful [57].

### 3.11. Ethical Considerations

A full ethics application was submitted to the Faculty of Medicine, Health and Life Sciences (MHLS) Research Ethics Committee (REC) at Queen’s University Belfast and ethical approval was granted in January 2025 (MHLS 24_135).

Informed consent process and confidentiality

Informed consent will be obtained from participants immediately prior to interviews taking place. Participants will be given an information sheet that clearly and sensitively outlines the study, including its aims, intended purpose, and methods; what their involvement would consist of; how their data would be collected, stored, and used; and the anticipated benefits and potential negative aspects of participating [51]. This information sheet will also prepare participants for the nature of the interview questions and advise them that they can withdraw from the study at any time and that doing so will not affect the care and support they (or their loved one) may be provided [58].

There will be two consent forms offered to participants: one for participation in interviews (with or without the use of photovoice) and one for dissemination of contributed photographs. It will be made clear to participants that they can participate in photovoice without releasing their photographs.

In relation to respecting participants’ confidentiality, data will be deidentified, analysed, retained and shared in accordance with the Declaration of Helsinki [59].

Distress protocol and participant support

Given that this will be research of a sensitive nature, it is anticipated that participants may experience emotions and distress whilst recalling and discussing their experiences. Therefore, the researcher interviewing the participant will utilise an agreed distress protocol based off Haigh and Witham’s [60], being mindful of not terminating an interview prematurely and negatively reinforcing participants’ vulnerability or inspiring feelings of rejection [61,62]. Questions that are sensitive in nature will be introduced in the middle of interviews to allow participants to ‘warm up’ to and ‘cool down’ from these topics. Data collection will occur in a comfortable environment at a time nominated by participants, but the primary researcher (LC) will ensure this is also when support services are available, in case they are required.

The researcher carrying out data collection (LC) will collaborate with our charity partner for participant support. This will involve giving participants a hardcopy sheet of signposted resources for further support [58], including contact information for local support, helplines, and e-resources [63,64]. There will also be a referral route available to participants for counselling if needed.

## 4. Discussion

### 4.1. Potential Implications of the Study

Establishing an in-depth understanding of people’s pancreatic cancer journeys and creating a robust theory to capture this will enable us to better understand and anticipate people’s needs when they are diagnosed with pancreatic cancer and contribute towards addressing the gaps in the literature on the experiences of people living with pancreatic cancer. A theory grounded in peoples’ lived experiences can inform our priorities for future care, service provision, research, and policy. For example, this theory could inform the implementation of the new regional Optimal Care Pathway for people with pancreatic cancer which has been launched in Northern Ireland [8] in recognition of the need for improvements to diagnosis, treatment, and care. This research could also contribute to the development of targeted support interventions to assist people to maintain the best possible QoL for as long as possible during their pancreatic cancer journey.

### 4.2. Anticipated Challenges and Limitations

We anticipate it will be more difficult to reach and recruit people whose pancreatic cancer is at a very advanced stage at diagnosis, due to how time-limited they may be. By speaking with bereaved care partners, we hope to gain insight into these experiences and ameliorate this limitation. Another anticipated challenge is that participants (particularly in Phase 2) might need to reschedule interviews at short notice if feeling unwell or due to caring responsibilities. Therefore, the primary researcher (LC) will ensure ease of contact via text message and telephone, emphasise flexibility in scheduling/rescheduling interviews, and monitor for signs of discomfort or fatigue during interviews. Limitations relating to the photovoice component are that people may not opt in due to potential discomfort with taking photographs or expressing themselves in this way, and the practicalities of sharing photographs with the primary researcher (LC), whether physical or using a digital device.

With regards to potential limitations associated with not using alternative data collection methods, dyadic interviews were considered for their ability to gather additional context and stimulate richer contributions. Yet, it was surmised that, due to the nature of the research, participants might instead be less candid in the presence of their spouses or other family if ‘putting a brave face on’ to shield them from aspects of their journeys [65,66,67].

Focus groups were also considered for Phases 2 and 3 as they could provide participants with a sense of peer support and solidarity. However, individual SSIs were ultimately chosen with participants’ wellbeing and welfare in mind. It is anticipated that participants may feel more comfortable sharing in-depth accounts of their personal experiences in a more private setting due to the sensitive nature of the topic and limits to confidentiality in a group setting [41,68,69,70,71]. We also factored in the potential hidden costs of planning and carrying out FGs, such as the difficulty in finding a convenient location for group participants and navigating everyone’s availability [72]. These considerations are especially relevant to Phase 2 and 3 participants. Phase 2 participants could need to reschedule at short notice if feeling unwell because of ongoing treatment, disease progression, or post-treatment symptoms, and they will likely feel more comfortable in their own home if experiencing symptoms relating to pancreatic exocrine insufficiency. Additionally, both Phase 2 and 3 participants might have caring responsibilities to consider. Ideally, we would conduct a focus group prior to individual interviews to generate broad data, explore this in more depth, and test concepts, but with these situations in mind, an individual interview in participant’ own homes (or local to them) was felt to be more appropriate and practical, and should limit no-shows.

### 4.3. Study Status

Recruitment is expected to begin in July 2025, with an estimated timeline for study completion by December 2026.

## Figures and Tables

**Table 1:** Resources required, and process followed for each phase of data collection.

Phase	Resources	Process Followed
**Phase 2:** SSIs with people diagnosed with pancreatic cancer	Consent forms Participant information sheet(s) Resources/signposting sheet for participants Interview topic guide Recording equipment: digital audio recorder and mobile phone as back-up NVivo 2nd transcriber/transcription software	Provide opportunity to ask questions. Obtain informed consent from participant (written, if possible; agree to recorded verbal if not). Start recording (gain oral consent if necessary). Use topic guide questions but provide space for participant to lead the interview, using probes as appropriate. If using photovoice, ask questions about the photograph and what it represents, probing as appropriate. Conclude interview and thank participants. Check audio recording of interview on digital recorder is working and upload to encrypted storage on OneDrive. Store consent form(s) in locked filing cabinet in secure office. Delete recording from audio recorder and backup on phone. Transcribe interview. Verify transcription. Review and analyse transcribed data.
**Phase 3:** SSIs with care partners of people diagnosed with pancreatic cancer	Consent forms Participant information sheet(s) Resources/signposting sheet for participants Interview topic guide Recording equipment: digital audio recorder and mobile phone as back-up NVivo 2nd transcriber/transcription software	Provide opportunity to ask questions. Obtain informed consent from participant (written, if possible; agree to recorded verbal if not). Start recording (gain oral consent if necessary). Use topic guide questions but provide space for participant to lead the interview, using probes as appropriate. If using photovoice, ask questions about the photograph and what it represents, probing as appropriate. Conclude interview and thank participants. Check audio recording of interview on digital recorder is working and upload to encrypted storage on OneDrive. Store consent form(s) in locked filing cabinet in secure office. Delete recording from audio recorder and backup on phone. Transcribe interview. Verify transcription. Review and analyse transcribed data.
**Phase 4:** FGs with professionals and advocates with insight into the experiences of people diagnosed with pancreatic cancer	Consent forms Participant information sheet(s) Resources/signposting sheet for participants Interview topic guide Recording equipment: digital audio recorder and mobile phone as back-up NVivo 2nd transcriber/transcription software 2nd moderator if four or more participants	Provide opportunity to ask questions. Obtain informed consent from participants (written, if possible; agree to recorded verbal if not). Start recording (gain oral consent if necessary). Use topic guide questions but provide space for participants to lead the interview, using probes as appropriate. If using photovoice, ask questions about the photographs and what they represent, probing as appropriate and encouraging balanced participation, moderating contributions. Conclude interview and thank participants. Check audio recording of interview on digital recorder is working and upload to encrypted storage on OneDrive. Store consent form(s) in locked filing cabinet in secure office. Delete recording from audio recorder and backup on phone. Transcribe interview. Verify transcription. Review and analyse transcribed data.

**Table 2:** Phase-specific inclusion/exclusion criteria for participants involved in data collection.

Phase	Inclusion Criteria	Exclusion Criteria
**All Phases**	Aged 18 and over.	
	Able to understand and speak English.	
	Able and willing to provide oral if not written consent to participate in the study.	
**Phase 2**	Living in NI whilst navigating pancreatic cancer journey. People who received care in other countries (for instance, for a procedure or surgery) will be included if their pancreatic cancer journey mostly took place in NI.	Pancreatic cancer journey predominantly took place outside NI.
	Pancreatic cancer of any stage and prognosis, current or historical diagnosis.	Advanced co-morbidities which are also life limiting, such as concurrent cancers, advanced heart failure, severe chronic obstructive pulmonary disorder, or other conditions that significantly impact life expectancy.
**Phase 3**	Have insight into the experiences of people who: (1) were diagnosed with pancreatic cancer in NI and (2) navigated their journey in NI.	
	Partners, former partners, family, friends, neighbours, and others who contribute(d) to the care of the person with pancreatic cancer and/or support(ed) them with decision-making, information gathering, emotional support, and communication [48,49]	
**Phase 4**	Have insight into experiences of people who: (1) were diagnosed with pancreatic cancer in NI and (2) navigated their journey in NI.	

## Data Availability

Deidentified and redacted data (transcripts and photographs) will be shared via the UK Data Service repository, since it is free and has access controls.

## Abbreviations

The following abbreviations are used in this manuscript:

ERG	Expert reference group
FG	Focus group
GT	Grounded theory
HPB	Hepato-pancreato-biliary
MHLS	Medicine, Health and Life Sciences
NHS	National Health Service
NI	Northern Ireland
NIPANC	Northern Ireland Pancreatic Cancer
QoL	Quality of Life
QUB	Queen’s University Belfast
SSI	Semi-structured interview
UK	United Kingdom
